# Ticks (Acari: Ixodidae) infesting cattle and some other domestic and wild hosts on the French Mediterranean island of Corsica

**DOI:** 10.1186/s13071-016-1876-8

**Published:** 2016-11-15

**Authors:** Sébastien Grech-Angelini, Frédéric Stachurski, Renaud Lancelot, Jérôme Boissier, Jean-François Allienne, Sylvain Marco, Oscar Maestrini, Gerrit Uilenberg

**Affiliations:** 1 INRA, UR045 Laboratoire de recherches sur le développement de l’élevage, 20250 Corte, France; 2 Corsican Health Research (CHR), 20111 Calcatoggio, France; 3 CIRAD, UMR CMAEE, 34398 Montpellier, France; 4 INRA, UMR 1309 CMAEE, 34398 Montpellier, France; 5 Université de Perpignan Via Domitia, IHPE UMR 5244, CNRS, IFREMER, Université de Montpellier, F-66860 Perpignan, France; 6 Université de Corse, 20250 Corte, France; 7 “A Surgente”, Route du Port, 20130 Cargèse, France

**Keywords:** Ticks (Ixodidae), Cattle, Domestic animals, Wild animals, Molecular identification, Corsica

## Abstract

**Background:**

Corsica is a mountainous French island in the north-western Mediterranean presenting a large diversity of natural environments where many interactions between domestic animals and wild fauna occur. Despite a favourable context for ticks and tick-borne diseases (TBDs), the tick fauna of Corsica has not systematically been investigated.

**Methods:**

For one year (May 2014-May 2015), a survey of ticks infesting cattle was performed in the three Corsican cattle slaughterhouses. Two of these were visited monthly and one quarterly; the ticks were manually collected, just after flaying. Ticks were identified on their morphology; when necessary, some specimens were also molecularly identified by sequencing mitochondrial *cox*1 (cytochrome *c* oxidase subunit 1) and 16S ribosomal RNA genes and ITS2 (internal transcribed spacer 2). During the same period, ticks from other domestic animals (small ruminants, horses, domestic carnivores) and wild animals (wild boars, mouflons, deer) were occasionally collected.

**Results:**

A total of 1,938 ticks was collected from 264 of 418 cattle examined, reared in 86 different localities. Eight tick species were found infesting cattle: *Rhipicephalus bursa* (56.1 %), *Hyalomma marginatum* (21.5 %), *Hy. scupense* (8.7 %), *Ixodes ricinus* (5.7 %), *Haemaphysalis punctata* (4.8 %), *Rh. sanguineus* (*sensu* *lato*) (2.3 %), *Rh.* (*Boophilus*) *annulatus* (0.7 %) and *Dermacentor marginatus* (0.2 %). The cattle infestation rate remained high all year (more than 50 %). Several tick species showed seasonal variation of their activity. From other Corsican animals 1,196 ticks were collected. Comparing ticks collected from cattle with those found on other animals, several host preferences were shown. A noteworthy record is that of a few *Ha. sulcata* on mouflons which were mainly infested by *Rh. bursa*.

**Conclusion:**

The Corsican tick fauna is characterized by typical Mediterranean species (*Rh. bursa* and *Hy. marginatum*), but the mild climate and diversified environment provide satisfactory habitats both for species usually found in dry areas (*Hy. scupense*) and species usually collected in humid areas (*I. ricinus*).

## Background

The importance of ticks lies not only in their direct effects (blood loss, damage to skins, debilitation), but usually even more in their role as vectors of pathogens. They may cause great losses to the livestock industry, particularly, but not only, in tropical and subtropical countries. Their regional importance depends to a great extent on the tick species and tick-borne pathogens (TBPs) present, but also on local climate, management and breeds of livestock [1]. It is also recognized that their role as vectors of human pathogens is second in importance after that of mosquitoes [2] and that they are worldwide the most important vectors in the veterinary field [3]. Human tick-borne diseases are usually zoonotic and wild and domestic vertebrate hosts are the reservoir of infection, usually without themselves being apparently affected [1].

**Fig. 1:**
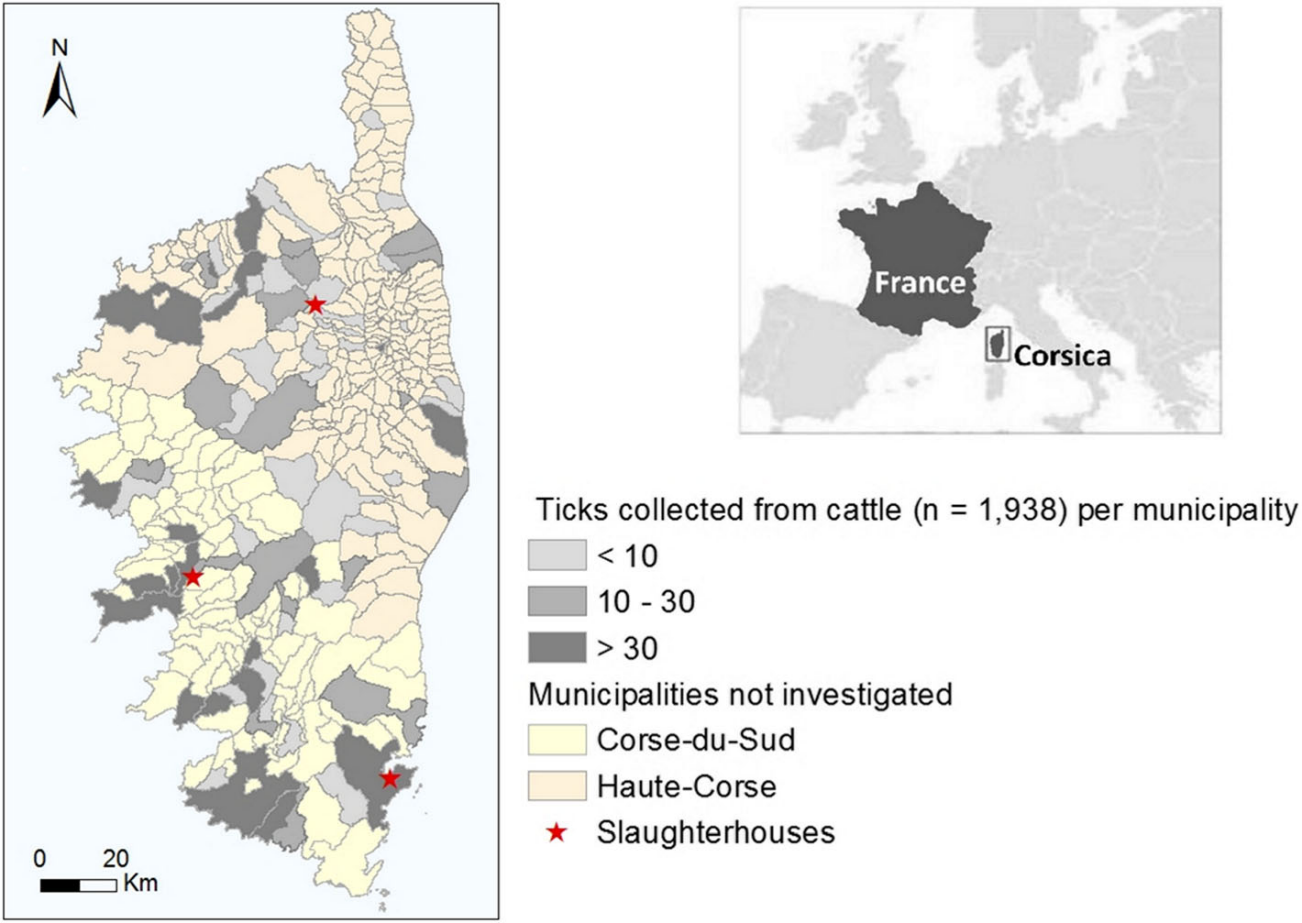
Distribution of the ticks collected from cattle in Corsica

Corsica is a French island in the western part of the Mediterranean area, situated 15 km north of Sardinia and 90 km west of Tuscany in Italy (Fig. 1). It is the fourth Mediterranean island in size (approximately 180 km long and 70 km wide) and also the most mountainous (with Monte Cintu peaking at 2,706 m) and the most forested one (46 % of the territory). Although its climate is considered as mild Mediterranean, there is some variability because of its specific geographical situation. Although Corsica is a sparsely populated region (about 320,000 inhabitants), about three million tourists visit the island annually. Hunting and hiking are very popular and livestock farming is an important economic activity (sheep, goats, pigs and cattle); it is of an extensive type and animals are often in contact with wildlife. Therefore in this context, important interactions occur between livestock, wildlife and humans in a small area, which certainly favours the circulation of TBPs, including zoonotic ones. However, neither the local tick fauna nor the pathogens they transmit have been systematically investigated.

There are approximately 70,000 cattle (mainly local *Bos taurus* used for meat production) and 1,000 cattle owners on the island. Cattle are reared over all the territory, they are kept outside all year and about 12,000 animals (mainly eight month-old) are slaughtered every year. This study reports the composition of tick fauna infesting cattle in Corsica and its variation in space and time. Some comparisons are made with ticks collected from other hosts. The discovery of *Hy. scupense* was reported in a previous paper [4].

## Methods

### Collection of ticks

Cattle were chosen as live traps because of the extensive, free-ranging livestock farming system, with a low frequency of acaricide treatments. Ticks were collected from May 2014 to May 2015 in the three Corsican cattle slaughterhouses (Ponte-Leccia in Haute-Corse, Cuttoli and Porto-Vecchio in Corse-du-Sud; Fig. 1). The slaughterhouses of Ponte-Leccia and Cuttoli were visited monthly, whereas samples were collected quarterly in Porto-Vecchio (in the extreme south of the island). During each visit, the whole skins of 15 animals on the average were examined, just after flaying, and the ticks were manually collected. When the number of ticks was low (less than 10), all were collected; otherwise between 10 and 15 were sampled for each animal.

The national cattle identification system, using ear-tags, allowed to trace the origin of animals at municipality level (the smallest administrative unit in France) and to determine the owner. By interviewing the farmers, the place where cattle were grazing could usually be determined. The altitude of their grassland was then estimated using Google Earth™.

**Fig. 2:**
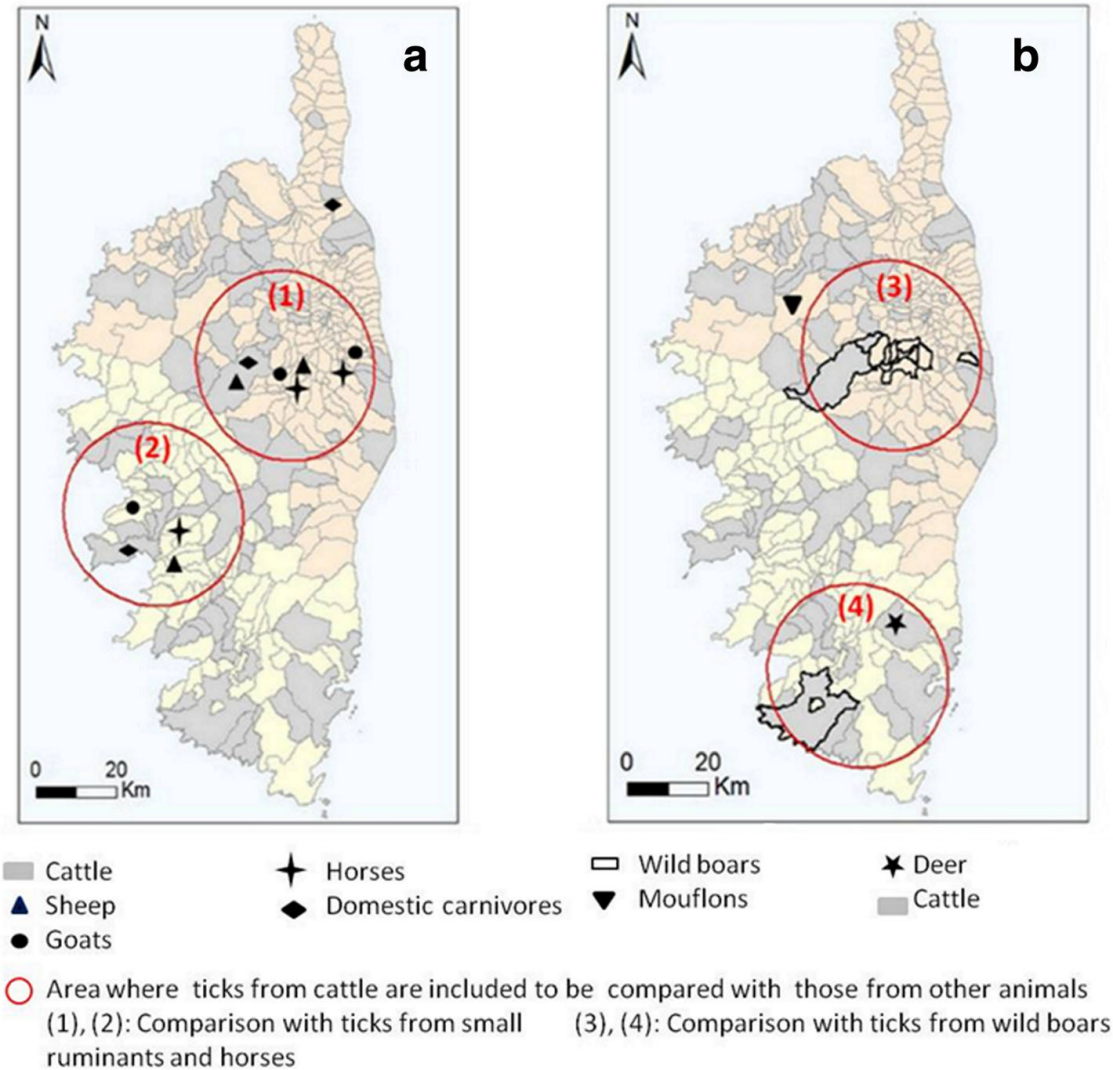
Localization of the ticks collected from domestic animals (**a**) and wildlife (**b**) and areas of comparison with ticks from cattle

Ticks from sheep, goats and horses were collected monthly from three farms (two in Haute-Corse and one in Corse-du-Sud) for each host species (Fig. 2a) from May to August 2014. At each visit, between 60 and 100 sheep or goats were inspected per farm while five horses were examined on the average in each riding school. Some of these animals (small ruminants and horses) were thus examined on different occasions. Practicing veterinarians provided ticks from dogs and cats during the same period.

Ticks from wild boars (*Sus scrofa*) were obtained from hunters during the hunting season (from August 2014 to February 2015) in two areas of Corsica (Fig. 2b). Staff of the National Office for Hunting and Wildlife (ONCFS) collected ticks from mouflons (*Ovis aries musimon*) from January to April 2015, and those of the Regional Natural Park of Corsica (PNRC) could obtain ticks from deer (*Cervus elaphus corsicanus*) in October 2014. To determine whether there was a host preference of tick species, ticks obtained from some domestic animals (goats, sheep and horses) and wild hosts (wild boars) were compared with ticks collected from cattle in the same area and during the same period (Fig. 2a, b). A circle of 40 km diameter was traced around the places where ticks from the interested hosts were collected; all the ticks collected from infested cattle reared inside these areas were included in the comparisons.

### Tick identification

After collection, ticks were stored in 70 % ethanol at −20 °C until they were identified under a stereomicroscope according to their morphological characteristics using appropriate keys and descriptions [5–8]. For some ticks species, never reported in Corsica or impossible to distinguish morphologically, molecular identification was attempted for a few specimens. Mitochondrial cytochrome *c* oxidase subunit 1 (*cox*1) and internal transcribed spacer 2 (ITS2) were used to identify *Hy. scupense*, as described in Grech-Angelini et al. [4]. There are great similarities between the different species of the *Rh. sanguineus* group [*Rh. sanguineus* (*s.l*.)] and for Nava et al. [9], it appears even impossible to define the species *Rh. sanguineus* (*sensu stricto*). The exact relationships between these species are under debate and, recently, a new phylogeny of the *Rh. sanguineus* group has been proposed using a 16S ribosomal marker [10]. This latter study, including several tens of 16S sequences from ticks recovered worldwide, proposes to split the *Rh. sanguineus* complex into four clades and nine Operational Taxonomic Units (OTUs). Subsequently, in order to position the Corsican specimens among the different OTUs, the same marker was used (and the same molecular methods) to distinguish tick species of *Rh. sanguineus* (*s.l*.), using nine specimens from different hosts. To improve these genetic analyses, three specimens of *Rh. bursa*, a tick species easily distinguishable based on morphological characters from other *Rhipicephalus* spp., were also genetically identified using mitochondrial 16S rDNA. Sequences obtained were compared with all sequences referenced in the study of Hekimoğlu et al. [10]. *Haemaphysalis sulcata* was morphologically identified in this survey; as it was very rarely collected in Corsica, it was also sequenced (one specimen of the four collected) and compared with the 16S sequence database. All genetic distances have been calculated using Mega 6.0. A Kimura-2parameters model of evolution was used, modifying the percent identity (see the results section) to give weight for different mutations according to either transitions or transversions.

### Data analysis

To assess differences in tick species distribution between host species, a beta-binomial logistic regression model was used to account for possible overdispersion in the response with respect to the binomial distribution [11, 12]. The response was the proportion of a given tick species, aggregated by host species, farm and sampling occasion. The dependent variable was the host species. A likelihood ratio test was used to assess the statistical significance of the host-species effect. Differences in tick species proportions were considered as statistically significant if *P* < 0.05.

## Results

### Cattle infestation

From May 2014 to May 2015, 418 cattle (3.5 % of the cattle slaughtered annually) from 161 farms (16 % of the Corsican farms) were inspected and 1,938 ticks were collected (56 % males, 33 % females and 11 % nymphs). An average of 2.6 bovines was inspected per farm. The sampled animals were reared in 86 communes and ticks were obtained from 74 of them (Fig. 1). The infestation rates were respectively 63 % for the cattle and 75 % for the farms. The cattle infestation rate varied from 71 % in May-June 2014 to 49 % in January-February 2015 (Table 1) whereas the infestation level ranged from one to more than 100 ticks per animal. Among the 264 infested cows, 46 % were infested by less than 10 ticks, 36 % by 10 to 30 ticks and 18 % by more than 30 ticks; almost 70 % of these high infestations were observed from March to June.

**Table 1:** Ticks collected, animals inspected and cattle infestation rate per 2 months period

Period	Ticks collected	Animals inspected	Infestation rate (%)
May-June (2014)	600	87	71
July-August	370	77	69
September-October	231	77	58
November-December	201	47	60
January-February (2015)	159	55	49
March-April	377	75	64
Total (over year)	1,938	418	63

### Species collected on cattle and molecular identification

Eight tick species were morphologically identified on cattle. The most abundant species were *Rh. bursa* (56.1 % of the ticks collected) and *Hy. marginatum* (21.5 %). *Hyalomma scupense*, *I. ricinus* and *Ha. punctata* were also frequently collected, whereas the other three species, i.e. *Rh. sanguineus* (*s.l*.), *Rh.* (*B.*) *annulatus* and *D. marginatus*, were much rarer (Table 2).

**Table 2:** Tick species collected from Corsican cattle

Species	No. of specimens	%
*Rhipicephalus bursa*	1,087	56.1
*Hyalomma marginatum*	417	21.5
*Hyalomma scupense*	168	8.7
*Ixodes ricinus*	110	5.7
*Haemaphysalis punctata*	94	4.8
*Rhipicephalus sanguineus* (*s.l*.)	45	2.3
*Rhipicephalus* (*B.*) *annulatus*	13	0.7
*Dermacentor marginatus*	4	0.2
Total	1,938	100

Concerning the molecular identification, two haplotypes (C1 and C2; see Table 3) were recorded for the mitochondrial 16S rDNA of the nine Corsican *Rh. sanguineus* (*s.l*.) analyzed. They clustered within two OTUs from nine recently identified OTUs [10]. Five ticks from cattle and three from dogs were identified as OTU9 (haplotype C1, KX553960), the genetic distance between the C1 haplotype and OTU9 being 1.4 % (54/399 bp). One tick collected from a dog was genetically close to the OTU4 (haplotype C2, KX553961); the genetic distance was 0.4 % (21/399 bp). The three specimens of *Rh. bursa* (C3, KX553962) showed a genetic distance of 0.4 % (25/399 bp) with those referenced by Hekimoğlu et al. [10]. With regards to *Hy. scupense,* which was never before identified on the island, its presence was confirmed by the molecular analyses; the genetic distance between the Corsican specimens and those deposited in GenBank was 0.7 % for both genes *cox*1 (59/497 bp) and ITS2 (10/1436 bp) [4].

**Table 3:** Pairwise genetic distances (in %) between mitochondrial 16S rDNA of reference sequences and Corsican specimens of the genus *Rhipicephalus* (C1, C2, and C3 sequences were compared with the whole database of Hekimoğlu et al. [10])

Species (morphology)	Sequence	*R. bursa*	C1	C2
*Rhipicephalus bursa*				
Corsican *Rhipicephalus sanguineus* (*s.l*.)	C1 (*n* = 8)	14.5 ± 2.1		
	C2 (*n* = 1)	11.3 ± 1.9	6.6 ± 1.5	
Corsican *Rhipicephalus bursa*	C3 (*n* = 3)	0.4 ± 0.2	13.8 ± 2.0	10.6 ± 1.8
*Rhipicephalus sanguineus* (*s.l*.)	OTU8	14.4 ± 2.1	6.1 ± 1.5	6.0 ± 1.3
	OTU5	16.6 ± 2.3	7.4 ± 1.4	6.7 ± 1.3
	OTU9	16.4 ± 2.5	1.4 ± 0.3	8.6 ± 1.8
	OTU1	14.4 ± 2.6	10.7 ± 2.1	9.3 ± 2.0
	OTU6	17.3 ± 3.2	9.0 ± 2.1	8.5 ± 2.0
	OTU7	15.9 ± 2.6	8.5 ± 1.8	7.6 ± 1.7
	OTU4	12.2 ± 2.0	7.7 ± 1.6	0.4 ± 0.2

**Fig. 3:**
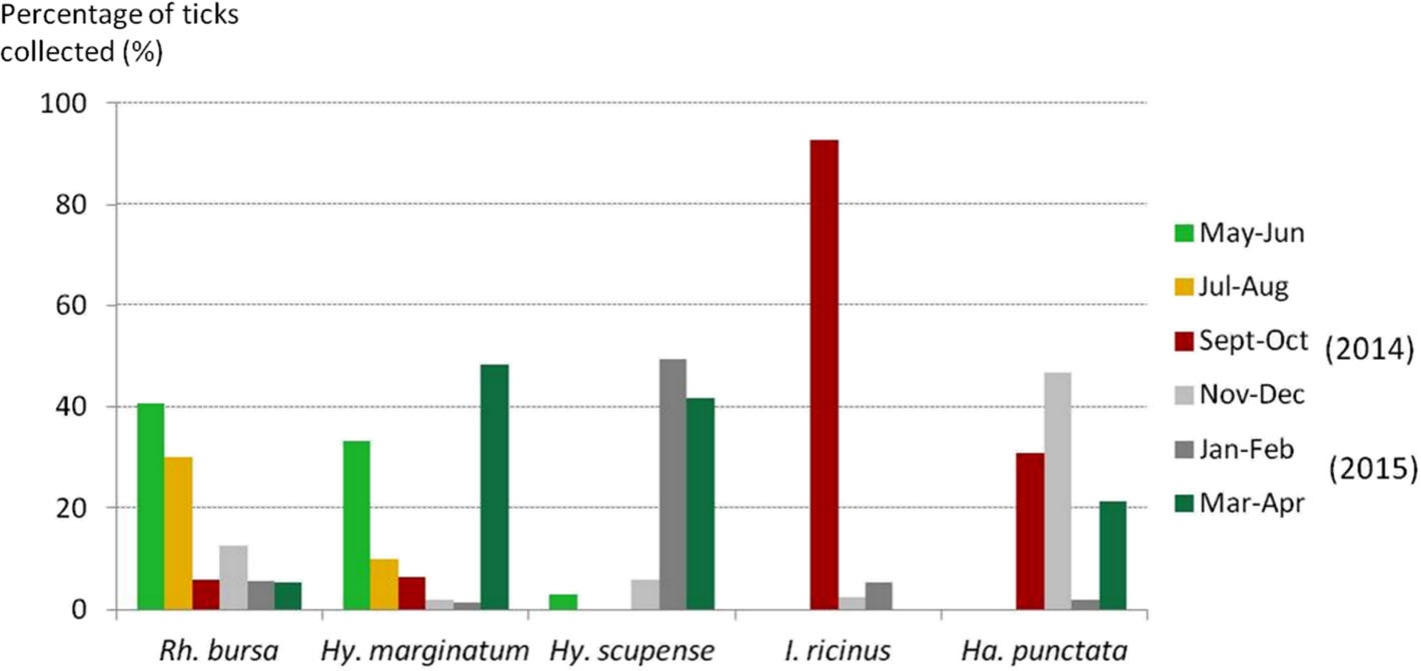
Seasonality of the main tick species found on Corsican cattle per 2 months

*Rhipicephalus bursa* and *Hy. marginatum* were collected throughout the year (Fig. 3). From May to August, *Rh. bursa* represented more than 70 % of the ticks collected from cattle. The activity of *Hy. marginatum* peaked from March to June when more than 80 % of the specimens were found; on the other hand, it was rarely collected from November to February. *Hyalomma scupense* was found from November to May with a peak activity from January to April when 90 % of the specimens were observed on cattle. More than 90 % of the *I. ricinus* were collected in September-October, representing more than 40 % of the ticks collected from cattle in that period (Fig. 3). *Haemaphysalis punctata* was found from September to April with a peak activity in November-December. *Rhipicephalus sanguineus* (*s.l*.) was collected from March to June and in September-October whereas *Rh.* (*B.*) *annulatus* was observed in July-August and from November to April. A few specimens of *D. marginatus* were collected from September to December.

**Fig. 4:**
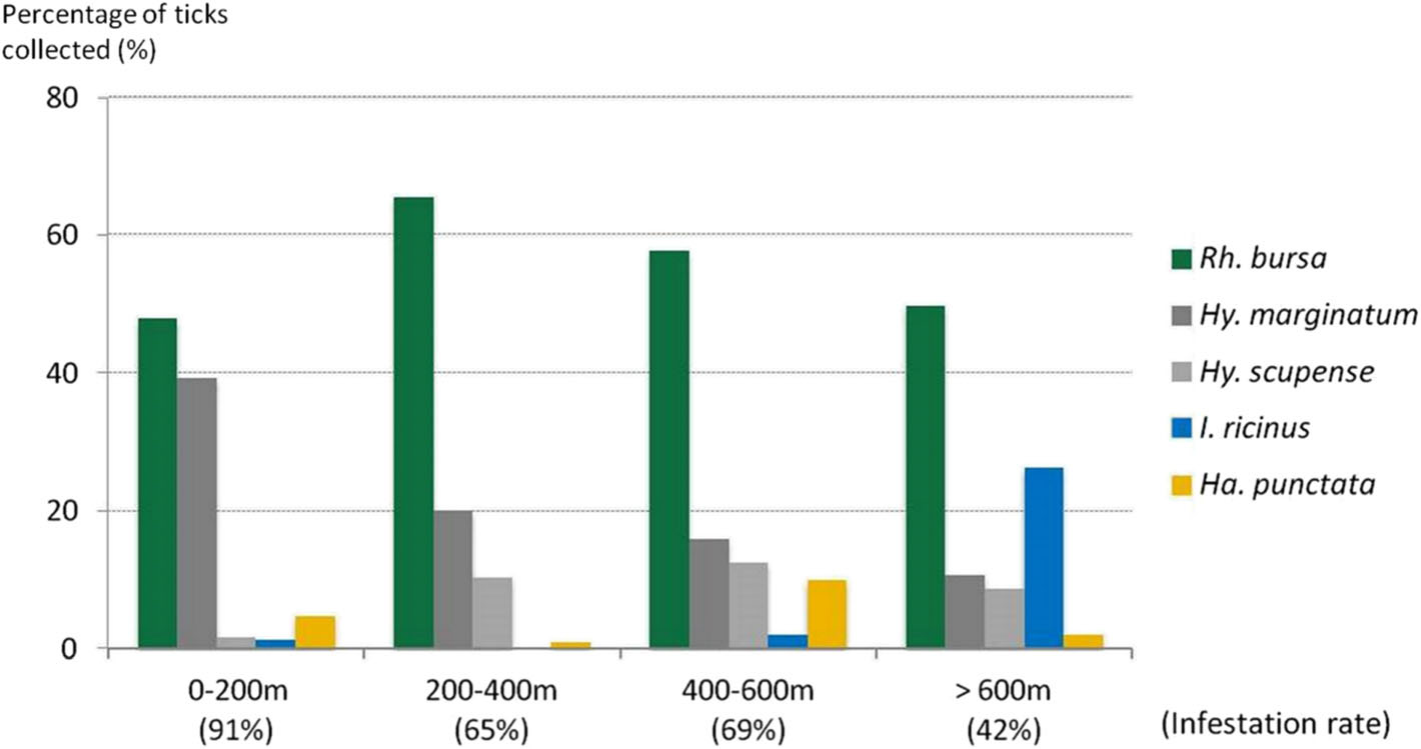
Percentage of the main tick species infesting cattle and cattle infestation rate according to altitude

Almost 60 % of the ticks were collected on cattle reared between 200 and 600 m above sea level. With the notable exception of *I. ricinus*, the infestation rate decreased with elevation: more than 90 % of the animals were infested by at least one tick below 200 m while only 42 % were infested over 600 m (Fig. 4). *Rhipicephalus bursa* was the main species collected at all altitude levels (Fig. 4). *Hyalomma marginatum* was much more frequently collected in areas with Mediterranean climate (at less than 200 m) representing almost 40 % of the ticks collected at this level, while more than 80 % of the *I. ricinus* were collected from cattle reared above 600 m.

### Comparison with other Corsican hosts

From May to August, 1,196 sheep and 908 goats were inspected. The infestation rates of sheep and goats were 4 and 28 %, respectively. *Rhipicephalus bursa* was by far the most abundant tick species on small ruminants, only a few specimens of other species having been collected from sheep and goats (Table 4). In the two areas where small ruminants were sampled (Fig. 2a) and during the same period (May to August), 267 ticks were collected from the 53 cattle examined, of which 71 % were *Rh. bursa* (98 % for sheep and goats). The observed proportion of *Rh. bursa* was significantly higher on small ruminants than on cattle (likelihood ratio test, *χ* ^2^ = 27.2, *df* =1, *P* < 0.0001). Among the 55 horses examined, 29 were infested by ticks (53 %). The most frequent tick species was *Hy. marginatum*, but *Rh. bursa* was also collected frequently (Table 4). Comparing tick species infesting horses with those collected from cattle (267 ticks collected from 53 animals) during the same period and in the same area (Fig. 2a), the observed proportion of *Hy. marginatum* was much higher on horses (78 %) than on cattle (26 %) (likelihood ratio test, *χ* ^2^ = 30.0, *df* =1, *P* < 0.0001). The domestic carnivores (31 infested dogs and four infested cats) were mainly infested by *Rh. sanguineus* (*s.l*.) (Table 4). One *I. ricinus* was collected from one cat and one *Hy. marginatum* from one dog.

**Table 4:** Ticks collected from other domestic animals

Host species (No. of infested animals)	Tick species	No. of specimens	%
Goats (258)	*Rhipicephalus bursa*	334	99.7
	*Haemaphysalis punctata*	1	0.3
	Total	335	100
Sheep (51)	*Rhipicephalus bursa*	53	91.4
	*Hyalomma marginatum*	4	6.9
	*Rhipicephalus sanguineus* (*s.l*.)	1	1.7
	Total	58	100
Horses (29)	*Hyalomma marginatum*	140	77.7
	*Rhipicephalus bursa*	38	21.1
	*Dermacentor marginatus*	1	0.6
	*Rhipicephalus sanguineus* (*s.l*.)	1	0.6
	Total	180	100
Domestic carnivores			
Dogs (31)	*Rhipicephalus sanguineus* (*s.l*.)	157	97.0
	*Hyalomma marginatum*	1	0.6
Cats (4)	*Ixodes ricinus*	1	0.6
	*Rhipicephalus sanguineus* (*s.l*.)	3	1.8
	Total	162	100

From 56 infested wild boars, hunted in the south and in the centre of Corsica (Fig. 2b), 297 ticks were collected (Table 5) of which *D. marginatus* represented almost 91 %. From cattle reared in the same area (and sampled during the same period), 310 ticks were collected from 78 animals of which less than 2 % were *D. marginatus*. This species infested significantly more wild boars than cattle (likelihood ratio test, *χ* ^2^ = 126.4, *df* =1, *P* < 0.0001). The 24 infested mouflons examined were mainly infested by *Rh. bursa* (Table 5). Four other species were observed on this host among which the species *Ha. sulcata* (*n* = 4), collected only on this wild ungulate. Its identification has been genetically confirmed (KX576650), the genetic distance between the Corsican specimen of *Ha. sulcata* and those deposited in GenBank (L34308.1) being extremely low: 0.6 % (17/359 bp). Ticks from some other wild animals were occasionally collected. *Ixodes ricinus* (*n* = 14) was the single species found on one deer examined in a mountainous area from southern Corsica (Fig. 2b). Migrating birds were caught in the northern part of the island and five of them were infested: three European greenfinch (*Chloris chloris*), one western yellow wagtail (*Motacilla flava*) and one common blackbird (*Turdus merula*). Four *I. ricinus* adults and one *Hy. marginatum* nymph were collected. Lastly, one hedgehog collected by hunters in central Corsica was infested by five adults of *Rh. sanguineus* (*s.l.*).

**Table 5:** Ticks collected from different Corsican wild ungulates

Host (No. of infested animals)	Tick species	No. of specimens	%
Wild boars (56)	*Dermacentor marginatus*	262	88.2
	*Rhipicephalus sanguineus* (*s.l*.)	20	6.7
	*Hyalomma marginatum*	8	2.7
	*Rhipicephalus bursa*	6	2.0
	*Ixodes ricinus*	1	0.4
	Total	297	100
Mouflons (24)	*Rhipicephalus bursa*	127	90.7
	*Haemaphysalis punctata*	7	5.0
	*Haemaphysalis sulcata*	4	2.9
	*Rhipicephalus sanguineus* (*s.l*.)	1	0.7
	*Dermacentor marginatus*	1	0.7
	Total	140	100
Deer (1)	*Ixodes ricinus*	14	100

### Spatial distribution of the main Corsican tick species

**Fig. 5:**
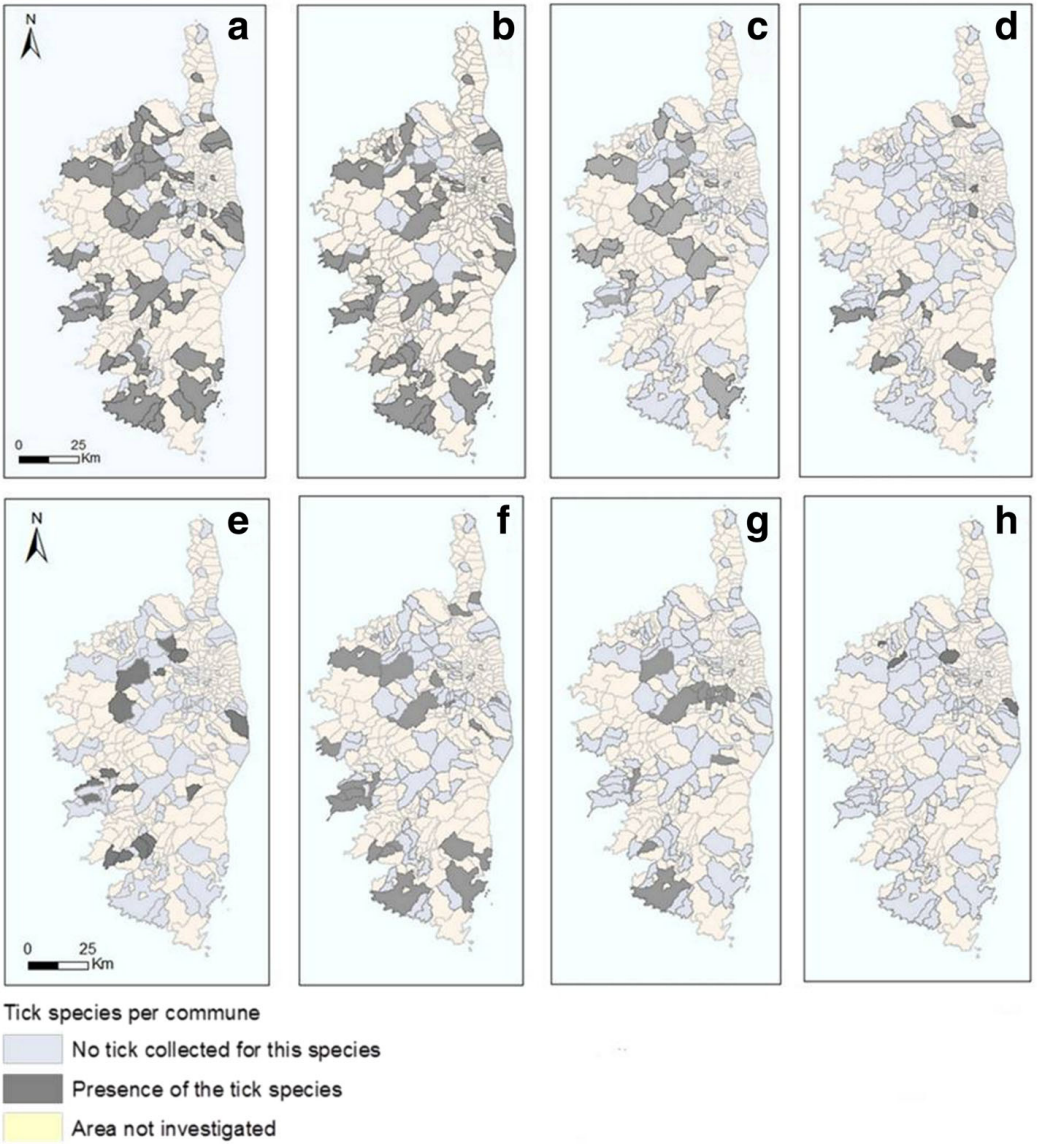
Distribution of the main Corsican tick species collected on animals. **a** *Rhipicephalus bursa*. **b** *Hyalomma marginatum*. **c** *Hyalomma scupense*. **d** *Ixodes ricinus*. **e** *Haemaphysalis punctata*. **f** *Rhipicephalus sanguineus* (*s.l.*). **g** *Dermacentor marginatus*. **h** *Rhipicepalus* (*Boophilus*) *annulatus*

Among the 3,134 ticks collected (more than 60 % from cattle), 53 % were males, 39 % females and 8 % nymphs. *Rhipicephalus bursa* and *Hy. marginatum* were spread over almost the entire investigated area (Fig. 5a, b). *Hyalomma scupense* was found mostly in Haute-Corse and especially in the centre of the island (Fig. 5c) where it could be very abundant [4]. *Ixodes ricinus* was identified in eight municipalities, and was the most collected species in some mountainous areas (Fig. 5d). *Haemaphysalis punctata* was mostly collected from cattle and it was found as well in southern as in northern parts of the island (Fig. 5e). *Rhipicephalus sanguineus* (*s.l*.) was dominant on dogs (Table 4), therefore the distribution area of these ticks was linked to dog sampling during the study (Fig. 2a); but they were also collected from cattle (in 13 municipalities) and wild boars (Fig. 5f). *Dermacentor marginatus* was collected almost only from wild boars and its spatial distribution is linked to the areas where these were sampled (Figs. 2b and 5g). *Rhipicephalus* (*B.*) *annulatus* was collected from cattle reared in four communes in Haute-Corse (Fig. 5h). The species *Ha. sulcata* (four ticks) was found only on mouflons in the commune of Asco (Haute-Corse), where the sampled animals lived (Fig. 2b).

## Discussion

Cattle were chosen as a model host to investigate the Corsican tick fauna because its husbandry system is still of a very extensive type: there are no barns, animals are reared outside all year round and acaricide treatments are performed without well-established criteria and with low frequency. Cattle may thus capture the tick species active in the environment and feeding on large mammals at all periods of the year. This choice was apparently efficient because the infestation rate of cattle was high throughout the year. From May to August 2014, ticks from small ruminants were also collected but cattle were much more infested during this period; it is probably due to the fact that sheep and goats are more handled, in particular during milking when breeders can check tick infestation every day. Moreover, eight of the nine tick species reported during the study were found on cattle; only *Ha. sulcata* was not collected from this host but from mouflons examined in a mountainous area of the island where no cattle were sampled (Figs. 1 and 2b). In much of continental France, according to the region, the tick species infesting most frequently cattle are *I. ricinus*, *D. marginatus*, *D. reticulatus*, *Ha. punctata* and *Rh. bursa* [8, 13]. This pattern is quite different from what was observed in Corsica. Regarding the geographical situation of the island, it appears more interesting to compare the Corsican tick distribution with other area of the Mediterranean basin and especially its islands.

*Rhipicephalus bursa* is a typical species in the Mediterranean area [6, 7] and was the most frequently collected species in Corsica. It was the dominant tick on cattle and small ruminants, as well as on mouflons and it was second in frequency on horses. This species is established in the whole of Corsica, both in coastal and mountainous areas. On Menorca Island, *Rh. bursa* was also the main tick species collected on cattle (more than 50 %) [14]; and it was frequently collected on small ruminants in Sardinia [15] and on cattle in Sicily [16]. *Rhipicephalus bursa* is also reported in North Africa, where it seems to be confined to the least dry areas of these arid or semi-arid regions [6, 7]. It is a proven vector of *Babesia bigemina* and *Babesia bovis* in cattle, and *Babesia ovis* in sheep; *B. bovis* has been detected in Corsica [17]. *Rhipicephalus bursa* is also a vector of *Anaplasma marginale* of cattle (also detected in Corsica [17]) and *Anaplasma ovis* of small ruminants, as well as of *Theileria equi* in equines [7].

The presence of *Hy. marginatum* in Corsica was reported by other authors [8, 18, 19], and confirmed by this survey. It appears to be common and wide-spread on the island, and was the second species in frequency on cattle and the dominant one on horses. The species was considered as rare in continental France [5, 8], but in the past few years numerous adults were found on horses in the south-eastern part of the country (near the town of Montpellier and in the Camargue region, [20]). It is also described in other Mediterranean islands and countries neighbouring Corsica. In Sicily, it represented 3.5 % of the ticks collected from cattle [16], 23 % in Sardinia [15] and 27 % in Menorca [14]. *Hyalomma marginatum* is also widely distributed in North Africa [7]. It is one of the main vectors of Crimean-Congo haemorrhagic fever (CCHF) virus, an important emerging zoonotic disease in Turkey and south-eastern Europe [21]. *Hyalomma marginatum* is a vector of *Rickettsia* spp. of the spotted fever group (SFG), including *R. aeschlimannii* in Corsica [19]. It has also been reported as a vector of equine babesiosis (caused by *Babesia caballi*) [7].

*Hyalomma scupense* (synonym *Hy. detritum*) was found for the first time on Corsica during this survey [4]. It was only collected on cattle, which is consistent with its host tropism, and appeared to be widespread and well-established (the third in frequency on Corsican cattle). This species was rarely found in the neighbouring areas. Very few specimens were recorded in Sardinia [22], on the small Italian island of Pianosa [23] and in continental Italy [24]. It was also reported, always in low numbers, in south-western continental France [25]. On the other hand, in North Africa *Hy. scupense* is often the main tick species infesting cattle [26]. This species is an efficient vector of tropical bovine theileriosis (caused by *Theileria annulata*), a major cattle disease in Northern Africa and a vector of *Theileria equi* and of *Coxiella burnetii*; it can probably also transmit CCHF virus but may not be a major vector [4].

*Ixodes ricinus* was considered as rare in Corsica [8], but in this survey represented almost 6 % of the ticks collected from cattle. It was mainly found in mountainous areas during autumn, when the conditions (temperature and humidity) were more favourable to this species. *Ixodes ricinus* was rarely collected in the dry areas and islands neighbouring Corsica. This species represented less than 1 % of the ticks collected from animals in Sicilia [16] and in Sardinia [15], but appears to be well-established in northern Italy where this tick is attaching frequently to humans [27]. In Africa, *I. ricinus* is restricted mainly to the coolest areas [7]; for instance, in the relative humid region of Gharb in northern Morocco, it represented 26 % of ticks collected from cattle [28]. This species is one of the main vectors of *Borrelia burgdorferi* (*s.l*.), the causative agent of Lyme disease, the most important zoonotic infection in Europe. This pathogenic agent was believed to be rare or even absent from Corsica, but a few cases of Lyme disease have been reported in recent years by the French Institute for Health Surveillance (InVS). *Ixodes ricinus* is also a vector of *Anaplasma phagocytophilum* (causative agent of tick-borne fever or pasture fever of ruminants), previously detected in bovine serum from Corsica [17]. The tick is also known as a vector of a number of other pathogens such as the zoonotic virus of tick-borne encephalitis (TBE), *Babesia divergens* (causative agent of bovine babesiosis), *Babesia venatorum* (human babesiosis), as well as *Rickettsia* spp. of the SFG [7, 8]. A related species, *Ixodes inopinatus*, has been described recently [29] from the drier regions of the western Mediterranean, and also as far north as Germany; it has been found to co-habit sometimes with *I. ricinus*. As this species was reported only from foxes and reptiles, not sampled in our survey, it has not been taken into consideration in this paper.

Almost 5 % of the ticks collected on cattle were *Ha. punctata*, found also in low numbers on mouflons and goats. This species is widespread in the entire Mediterranean basin and further to the north; it is found in a very wide variety of habitats [7]. It is a vector of *Theileria buffeli*, usually a benign blood parasite of cattle, which is known to occur on Corsica [17]. *Haemaphysalis pucntata* is also the vector in Europe of protozoa of the genus *Babesia*, *B. major* and *B. motasi*, causing (usually benign) babesiosis in cattle and small ruminants, respectively [7].

*Dermacentor marginatus* was very rarely collected on Corsican cattle and almost all specimens were found on wild boars. Cattle and wild boars commonly share the same areas of forest or maquis (sclerophyll shrubland) in Corsica. The comparisons between the ticks collected from cattle reared in the zones where wild boars were hunted (Fig. 2b), showed a pronounced host preference of *D. marginatus* for this wild ungulate. It is a common tick in the Mediterranean region [7]. In Sardinia, *D. marginatus* also infested mainly wild boars [30] and in Sicily, this species represented 1.4 % of the ticks collected from cattle [16]. In continental Spain, *D. marginatus* represented 9.3 % of the ticks collected on wild boars [31] but it was not reported on Menorca where wild boars are not present [14]. In Northern Africa, its distribution area is restricted to the coolest and most humid areas. *Dermacentor marginatus* can transmit various pathogens, including *Theileria equi* and *Babesia caballi* to horses (two causative agents of equine piroplasmosis), *Anaplasma ovis* to sheep, and also *Rickettsia slovaca* which belongs to the SFG [7, 8].

*Rhipicephalus sanguineus* (*s.l*.) ticks were found in all areas where domestic carnivores (especially dogs) were sampled. They have also been collected on cattle, mouflons, wild boars and more rarely on sheep and horses. Because of the strong association with domestic dogs, *Rh. sanguineus* (*s.l*.) ticks occur in all climatic regions of the Mediterranean basin. Among other pathogens, *Rh. sanguineus* (*s.l*.) is well known to transmit *Ehrlichia canis* and *Babesia vogeli* (causing respectively canine ehrlichiosis and canine babesiosis), *Babesia ovis*, *Anaplasma marginale* (these two latter diseases are known to occur in Corsica [17]) and *Anaplasma ovis* [7]. It is also a vector of rickettsiae of the SFG including *R. conorii*, the classical agent of human Mediterranean spotted fever; and its role as a vector of *R. massiliae* has been shown in Corsica [32].

*Rhipicephalus* (*Boophilus*) *annulatus* had already been identified in Corsica [33] but it has never been reported in continental France. This species was not frequently collected during this survey, infesting only cattle and representing less than 1 % of the ticks found on this host. In some other Mediterranean areas, *Rh.* (*B.*) *annulatus* is more common: on Menorca Island, it was the third species collected on cattle [14] and the main species collected from cattle reared in the northern part of Algeria [34]. This species is known as a vector of several pathogens, including *Babesia bovis*, *Babesia bigemina* and *Anaplasma marginale* [7].

*Haemaphysalis sulcata* (syn. *Ha. cretica*) has been reported only once in Corsica in a rather vague way in 1927 [35]. Its presence, confirmed by molecular identification, was not unexpected because it is a common tick in the Mediterranean region. It was reported in Sardinia on small ruminants [30], but also in Sicily, continental Italy, continental France, Spain and Northern Africa with lower frequency [7]. In Corsica, the species was only collected from mouflons in the northern part of the island. *Haemaphysalis sulcata* is known to transmit *Anaplasma ovis* to sheep.

## Conclusions

In this study 1,938 ticks were collected from cattle for 1 year. Eight tick species belonging to five genera were found. The pattern of the Corsican tick fauna infesting cattle is characterized by the dominance of typical Mediterranean species (*Rh. bursa* and *Hy. marginatum*) and by the existence of established populations of tick species usually found in drier areas (*Hy. scupense*), or in cooler areas (*I. ricinus*). Ticks collected from other domestic animals and wildlife confirmed that *Rh. bursa* and *Hy. marginatum* are the two most important tick species infesting ungulates in Corsica, and also confirmed the occurrence of another tick species, *Ha. sulcata,* found on mouflons. This first systematic survey of the tick fauna on Corsican livestock revealed important differences in composition and seasonal variation of the activity of some species with the situation of the Mediterranean regions neighbouring the island. It is now of the utmost importance for human and animal health to determine TBPs present in Corsica.

## Abbreviations

CCHF: Crimean-Congo haemorrhagic fever
*cox*1: Cytochrome *c* oxidase subunit 1
ITS2: Internal transcribed spacer 2
ONCF: National Office for Hunting and Wildlife
OTUs: Operational taxonomic units
PNRC: Regional Natural Park of Corsica
TBDs: Tick-borne diseases
TBPs: Tick-borne pathogens
